# The *FCGR3A* 158 V/V-genotype is associated with decreased survival of renal allografts with chronic active antibody-mediated rejection

**DOI:** 10.1038/s41598-021-86943-3

**Published:** 2021-04-12

**Authors:** Nicolle Litjens, Annemiek Peeters, Judith Kal-van Gestel, Mariska Klepper, Michiel Betjes

**Affiliations:** Department of Internal Medicine, Section Nephrology and Transplantation, Na516, Erasmus MC, University Medical Center, P.O. Box 2040, 3000 CA Rotterdam, The Netherlands

**Keywords:** Translational immunology, Transplant immunology

## Abstract

Natural killer (NK) cells express the Fc-gamma receptor CD16 (*FCGR3A*) and could therefore mediate renal endothelial cell damage in cases of chronic-active antibody mediated rejection (c-aABMR). The V/V-genotype of the *FCGR3A* 158 F/V polymorphism is associated with increased CD16 expression and cytotoxicity by NK cells. This study evaluated whether this genotype is associated with the diagnosis of c-aABMR and renal allograft loss. The distribution of the *FGCR3A* 158 F/V-genotypes was not different for c-aABMR cases (N = 133) compared to control kidney transplant recipients (N = 116, P = 0.65). The V-allele was associated with increased median fluorescence intensity (MFI) of CD16 by NK cells (MFI 3.5 × 10^4^ versus 1.3 × 10^4^ for V/V and F/F-genotype, P < 0.001). Increased expression of CD16 correlated with CD16-dependent degranulation of NK cells (R = 0.4; P = 0.02). Moreover, the V/V-genotype was significantly associated with a higher glomerulitis score and an independent risk factor (HR 1.98; P = 0.04) for decreased allograft survival. Death-censored graft survival in c-aABMR cases at 3 years follow-up was 33% for the *FCGR3A* 158 V/V-genotype versus 62% for the F/F-genotype. In conclusion, the *FCGR3A* V/V-genotype increases CD16-mediated NK cell cytotoxicity and is associated with a higher glomerulitis score and decreased graft survival in cases with c-aABMR.

## Introduction

Long-term allograft survival has shown little improvement over the last decades. An important factor compromising long-term allograft survival in kidney transplantation is chronic-active antibody mediated rejection (c-aABMR)^1,2^. The histomorphological lesions of c-aABMR develop over time and are associated with recurrent and episodic endothelial cell activation caused by immunoglobulin G (IgG)-antibodies recognizing donor-specific human leukocyte antigens (HLA) (DSAs) expressed on renal endothelial cells. Antibodies can also be formed against non-HLA antigens^3^. Both complement-dependent and -independent mechanisms have been proposed to contribute to the DSA-mediated graft injury^4^. Complement-independent mechanisms involve antibody-mediated cellular cytotoxicity (ADCC) exerted by γδ T cells, polymorphonuclear neutrophils (PMN) or natural killer (NK) cells through interaction of Fc gamma receptors (FCGRs) with DSAs bound to endothelial cells^5–7^. These FCGRs differ in IgG affinity and cellular distribution and signaling mechanisms. There are two types of FCGRs, inhibiting and activating. Amongst activating FCGRs, 4 have low (FCGR2A, FCGR2C, FCGR3A and FCGR3B) and 1 high IgG-binding affinity (FCGR1)^5^. Evaluation of transcriptomic signatures in renal allograft biopsies have revealed *FCGR3A* transcripts to be enriched which correlated with DSAs and ABMR. Together with enrichment of other NK-associated transcripts this supported the role of antibody-dependent cellular cytotoxicity (ADCC) in chronic rejecting renal allografts^8–10^. In addition, increased expression of the FCGR3A (known as CD16) was noted on circulating NK cells of kidney transplant recipients with a diagnosis of c-aABMR^11^ and markers that reflect CD16-dependent activation of circulating NK cells may identify heart transplant recipients at risk for developing cardiac allograft vasculopathy^12^.

Genetic variation in *FCGR* genes may affect susceptibility to antibody-mediated rejection. A single nucleotide substitution within *FCGR3A* gene results in allelic variation in amino acid 158 (phenylalanine-F or valine-V) in the IgG binding domain of the CD16 receptor, which impacts the expression of CD16 and antibody-dependent cellular cytotoxicity (ADCC) function of NK cells.

After diagnosis of c-aABMR progression to renal allograft failure and response to therapy is highly variable and not readily explained by clinical characteristics and Banff scores^13–15^. In addition, some patients have only subclinical c-aABMR, which is only detected by a protocol biopsy. A recent study by Arnold et al.^16^, showed the V-allele of this *FCGR3A* 158 F/V single nucleotide polymorphism (SNP) to be associated with a higher level of microvascular inflammation in a cohort of DSA-positive kidney transplant recipients diagnosed with ABMR. The aim of our study was to evaluate whether the V/V-genotype of this *FCGR3A* 158 F/V SNP might identify kidney transplant recipients at higher risk to have a clinical relevant c-aABMR and may influence the rate of of renal allograft loss.

## Results

### Characteristics of the study population

This study included 133 kidney transplant recipients, diagnosed with c-aABMR. The baseline characteristics (prior to kidney transplantation) as well as those at time of biopsy of the study population is given in Table 1. The median age at time of transplantation was 46 (IQ range 23) years. The majority of patients received a kidney from a living donor and 37 patients were retransplanted. Eleven transplantations were ABO-incompatible (ABOi). ABOi kidney transplantations are not associated with inferior long-term graft survival or increased incidence of c-aABMR^17^ and were therefore not excluded from the study cohort. A for-cause biopsy was performed at a median of 5.3 years after transplantation and most kidney transplant recipients were on dual immunosuppression (72%), receiving tacrolimus-based immunosuppression. All c-aABMRh cases received the combination of IVIG and methylprednisolone, which is the standard treatment for c-aABMR in our center^15^. The median (interquartile range, IQR) eGFR at time of diagnosis was 34 (18) mL/min/1.73 m^2^.

**Table 1:** Demographic and patient characteristics at baseline and time of diagnosis of c-aABMRh.

Parameters	Kidney transplant recipients diagnosed with c-aABMRh				P value
	Total (N = 133)	V/V genotype (N = 21)	F/V genotype (N = 59)	F/F genotype (N = 53)	
**At time of transplantation**					
Recipient age (years)	46 (23)	47 (21)	46 (21)	46 (23)	1.00
Donor age (years)	50 (16)	50 (11)	49 (18)	52 (19)	0.85
Recipient gender (male)	58%	57%	53%	64%	0.46
Deceased donor	30%	38%	24%	34%	0.34
ABO-incompatible	8%	5%	10%	6%	0.58
Re-transplantation	28%	33%	20%	34%	0.23
HLA mismatch in A, B, and DR	3 (3)	4 (3)	3 (2)	3 (3)	0.24
Current PRA	0 (5)	0 (8)	0 (7)	0 (4)	0.78
Historic PRA	4 (48)	5 (28)	5 (43)	4 (61)	0.96
Induction therapy, N (%)					0.001
Basiliximab	43 (33%)	5 (21%)	18 (31%)	20 (38%)	
Anti-thymocyte globulin	5 (4%)	0 (0%)	0 (0%)	5 (9%)	
Pre-transplant immunoadsorption	10 (8%)	1 (5%)	5 (8%)	4 (8%)	
**At time of biopsy**					
Maintenance immunosuppression, N (%)					0.01
Triple immunosuppressio n	29 (22%)	3 (14%)	9 (15%)	17 (32%)	
Dual immunosuppressio n	96 (72%)	17 (81%)	45 (76%)	34 (64%)	
Single immunosuppression	8 (6%)	1 (5%)	5 (9%)	2 (4%)	
Immunosuppressive agents, N (%)					
Tacrolimus	112 (84%)	18 (90%)	48 (81%)	46 (87%)	
Cyclosporin A	2 (2%)	0 (0%)	2 (3%)	0 (0%)	
mTOR inhibitor	9 (7%)	2 (10%)	3 (5%)	4 (8%)	
MPA or azathioprine	120 (90%)	20 (95%)	52 (88%)	48 (91%)	
Steroid	45 (34%)	4 (19%)	17 (29%)	24 (45%)	
DSAs	70	13	28	29	
Positive	28 (40%)	7 (54%)	9 (32%)	12 (41%)	0.41
Negative	42	6	19	17	
C4d in PTC	113	18	49	46	
Positive	24 (21%)	3 (17%)	9 (18%)	12 (26%)	0.57
Negative	89	15	40	34	
eGFR (mL/min/1.73 m^2^)^a^	34 (18)	29 (18)	35 (15)	35 (18)	0.11
Time to biopsy (years)	5.3 (5.6)	2.7 (5.1)	5.4 (4.8)	5 (6.2)	0.37
Recipient age (years)	53 (19)	52 (17)	52 (20)	54 (21)	0.96

Data represent median (IQR) and number (proportion of total), respectively.

^a^Estimated glomerular filtration rate (eGFR): calculated using the CKD-EPI formula, multiplied by 1.159 when a patient has the African/Caribbean ethnicity.

### The *FCGR3A* 158 V/V-genotype is not a risk factor for developing c-aABMR after kidney transplantation

To evaluate whether the *FCGR3A* 158 FV/V-genotype could stratify kidney transplant recipients at risk for developing c-aABMR, we included a cohort of kidney transplant recipients, transplanted within the same period, but without a diagnosis of c-aABMR. As ethnicity was found to influence the distribution of many *FCGR* polymorphisms^18,19^, we compared the distribution of ethnicity (P = 0.85), which was similar for both groups. The proportion of male kidney transplant recipients was not different for both groups (P = 0.31). The c-aABMRh group was younger than control population (P = 0.04), i.e. the median (IQR) age amounted to 46 (23) years and 50 (20) years, respectively.

Genotyping for *FCGR3A* 158 F/V polymorphism was performed using gene-specific primers and PCR amplification^20^ as briefly described in Materials and Methods section. The genotype frequencies of *FCGR3A* 158 F/V are shown in Table 2. The genotype distribution did not deviate from the expected genotype frequencies at the Hardy–Weinberg equilibrium, calculated from allele frequencies in both cohorts (Table 2). Moreover, no significant difference (P = 0.65) was observed for the distribution of the *FCGR3A* 158 F/V genotypes between c-aABMRh and control kidney transplant recipients. Frequencies amounted to 16% versus 15%, 44% versus 40% and 40% versus 46% with respect to the V/V, F/V and F/F-genotypes for c-aABMRh and control kidney transplant recipients, respectively. In summary, the *FCGR3A* V/V-genotype does not identify kidney transplant recipients at risk for developing c-aABMR following kidney transplantation.

**Table 2:** *FCGR3A* 158 F/V-genotype frequencies.

Genotype	Kidney transplant recipients				*P* value
	c-aABMRh (N = 133)		Controls (N = 116)		
	Observed (N, %)	Expected (N, %)^a^	Observed (N, %)	Expected (N, %)^a^	c-aABMRh^+^ vs. control
V/V	21 (15.8)	23 (17.7)	17 (14.7)	20 (16.8)	0.65
F/V	59 (44.4)	65 (48.7)	46 (39.7)	56 (48.4)	
F/F	53 (39.8)	45 (33.6)	53 (45.7)	40 (34.8)	
*P* value (observed vs. expected)	0.60		0.22		
**Allelic frequency**					
V_158_	80 (42)		63 (41)		
F_158_	112 (58)		99 (59)		

^a^Expected genotype frequencies at Hardy Weinberg equilibrium were calculated from allele frequencies in c-aABMRh and control subjects.

### The V-allele of *FCGR3A* is associated with increased CD16 expression and CD16-mediated NK cell function

**Figure 1:**
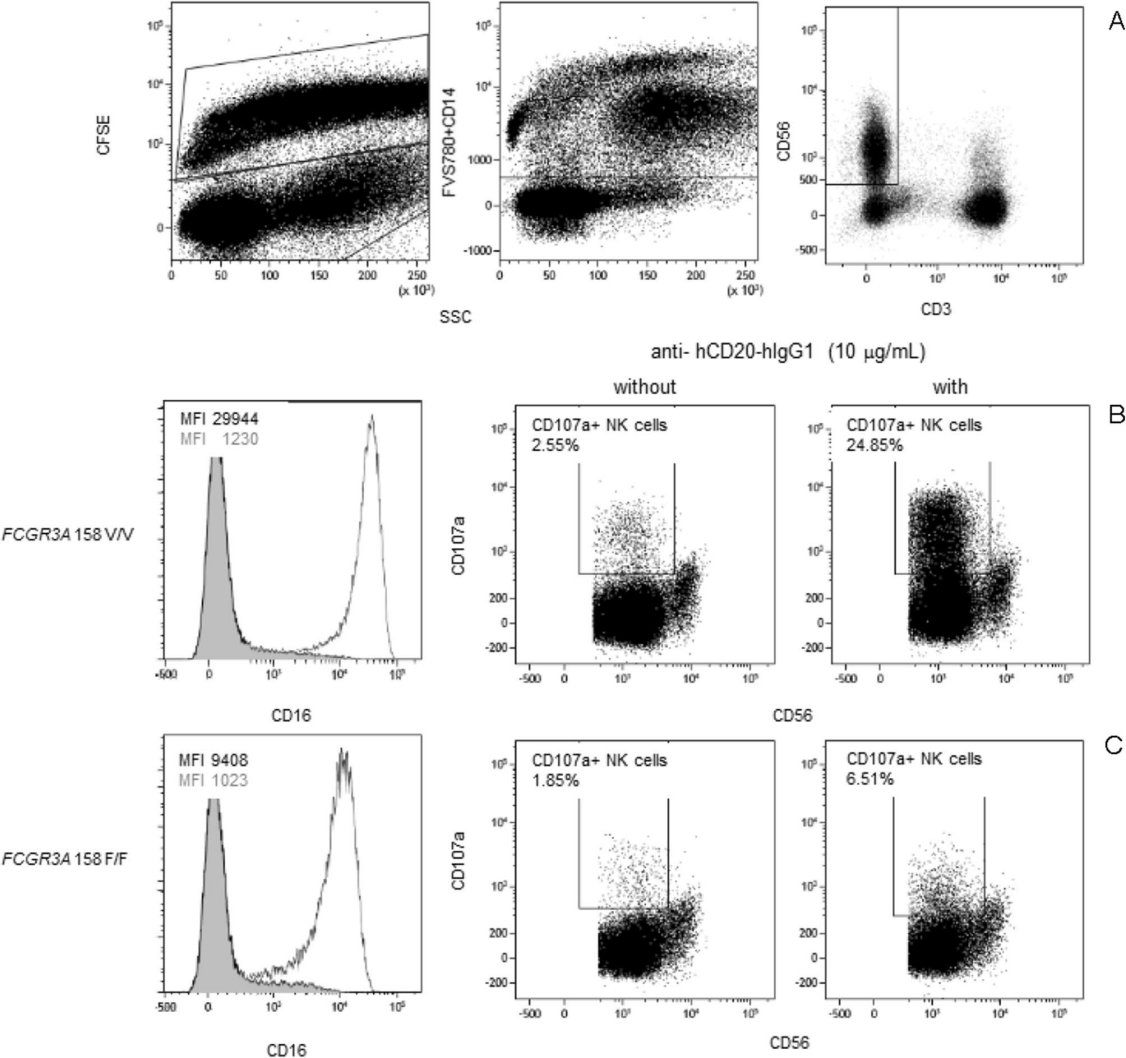
*FCGR3A* 158 F/V -genotype and flowcytometric analysis of CD16 expression and CD16-dependent NK cell function. A typical example of the flowcytometric analysis of CD16 expression and CD16-mediated NK cell function is depicted for PBMCs co-cultured with CFSE-labeled Raji (target cells). Briefly, viable CD56 + NK cells were identified within CFSE-negative PBMCs (**A**, left plot) by excluding dead cells, cells expressing either CD14 (**A**, middle plot) and CD3 (**A**, right plot). Median fluorescence intensity of CD16 as well as percentages of CD107a-expressing NK cells were depicted for a kidney transplant recipient having the V/V- (**B**) and F/F-genotype (**C**). The histograms illustrate CD16 downregulation following co-culture in presence of 10 μg/mL anti-hCD20-hIgG1, with the open and grey histograms reflecting the expression in absence and presence of anti-hCD20-hIgG1, respectively. The middle dotplots (**B**,**C**) depict the expression of CD107a (percentage) in absence of and those on the right (**B**,**C**) reflect that in presence of anti-hCD20-hIgG1, respectively.

**Figure 2:**
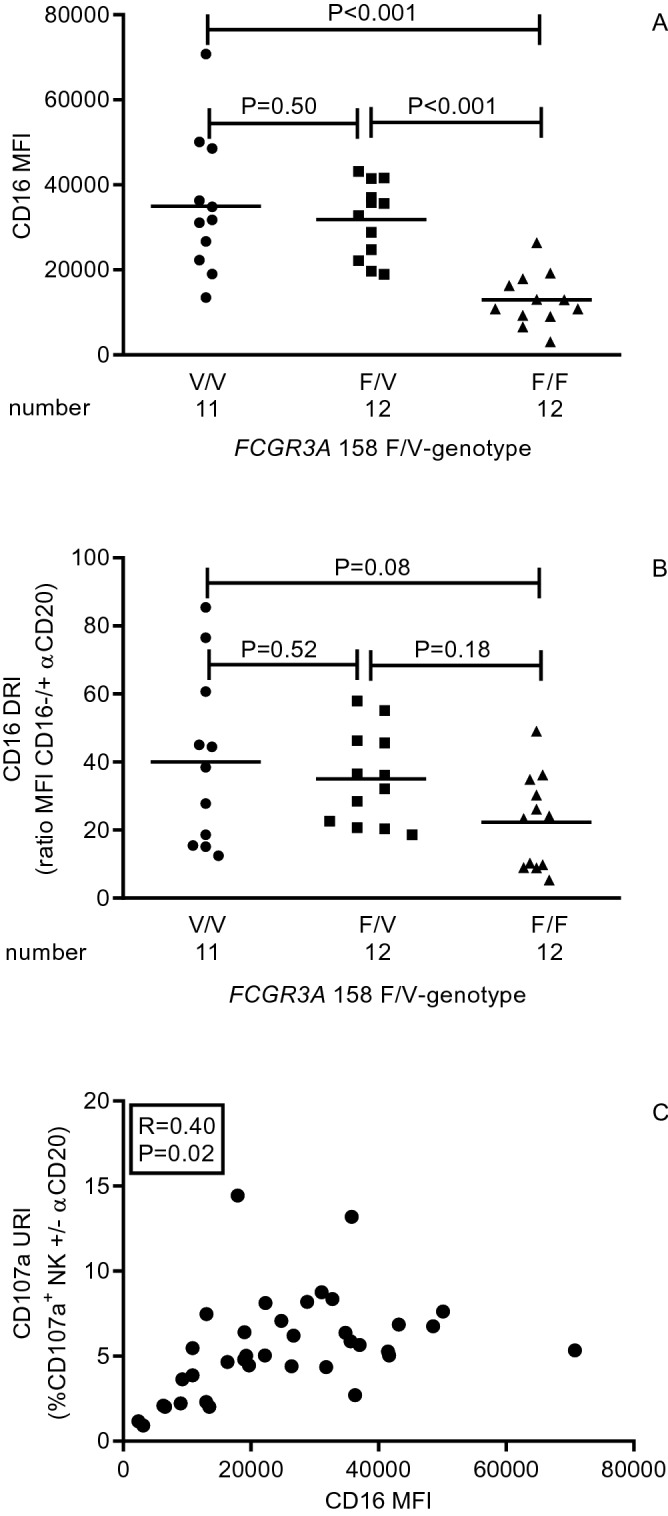
*FCGR3A* 158 F/V-genotype and CD16-dependent NK cell function. Median fluorescence intensity (MFI) of CD16 by NK cells (**A**) were measured in 35 samples genotyped for *FCGR3A* 158 F/V. The anti-hCD20-hIgG1-mediated downregulation of CD16 was measured for 35 samples genotyped for *FCGR3A* 158 F/V (**A**). Circles represent V/V-genotype, squares the F/V-genotype and triangles the F/F-genotype. The horizontal line represents the mean value per genotype. The baseline MFI of CD16 on circulating CD16^+^ NK cells, displayed on the X-axis, was associated to the anti-hCD20-hIgG1-mediated upregulation of CD107a, displayed on the Y-axis for these 35 samples (**C**). Differences between the genotypes were analyzed by the parametric one way Anova, followed by the Bonferroni’s multiple comparison test to determine where significant differences were present. P values < 0.05 were considered statistically significant.

Next, we assessed whether the *FCGR3A* 158 F/V-genotype is related to the phenotype and function of circulating NK cells by assessing the median fluorescence intensity (MFI) of CD16 on circulating NK cells and CD16-mediated NK cell function expressed as CD16 downregulation index (CD16 DRI) and CD107a upregulation index (CD107a URI). The gating strategy (Fig. 1A) as well as a typical flowcytometric example for a kidney transplant recipient having the V/V-genotype (Fig. 1B) and F/F- genotype (Fig. 1C) are depicted in Fig. 1. The MFI of CD16 on NK cells was significantly higher (P < 0.001) for the V/V-genotype and F/V-genotype when compared to the F/F-genotype (Fig. 2A). Mean MFI for CD16 were 3.5 × 10^4^ and 3.2 × 10^4^ and 1.3 × 10^4^ for the V/V-, F/V- and F/F-genotype, respectively. Presence of anti-human CD20-human-IgG1, resulted in downregulation of CD16 on circulating NK cells and *FCGR3A* 158 F/V-genotypes tended to be associated with the CD16 DRI (R = − 0.31; P = 0.06). Mean (95% CI) CD16 DRI amounted to 40.0 (22.9–57.1), 35.1 (26.3–43.8) and 22.3 (13.5–31.0) for the V/V-, F/V- and the F/F-genotype, respectively (Fig. 2B). In addition, the CD16 MFI of NK cells was positively associated (Pearson’s correlation coefficient; R = 0.40; P = 0.02) with the CD16-dependent degranulation potential of NK cells, expressed as CD107a URI (Fig. 2C). Summarizing, our results revealed the V-allele to be associated with an increased expression of CD16 on circulating NK cells and CD16-dependent NK cell function.

### The V-allele of *FCGR3A* is associated with renal microvascular inflammation

The *FCGR3A* 158 F/V-genotypes were related to degree of microvascular inflammation [MVI] as measured by the Banff scores for glomerulitis (g) and peritubular capillaritis (ptc). Cases having the V-allele had significantly higher g-scores at time of renal allograft biopsy. The median g-score amounted to 2, 2 versus 1 for the V/V-, F/V- versus F/F-genotype (P = 0.01), respectively. In early ABMR but not caABMR^14^, presence of DSAs were found to be associated with extent of microvascular inflammation^21^. We evaluated whether the presence of DSAs was associated with the degree of renal microvascular inflammation in cases with c-aABMRh in the this cohort. Although, the degree of MVI tended to be associated with presence of DSAs (P = 0.09), no significance was reached for the association with g (P = 0.27) or ptc (P = 0.68) scores.

Only 28 out of 70 cases tested for DSAs were DSA-positive (Table 1), which limits the evaluation of association with *FCGR3A* 158 F/V-genotypes with level of inflammation for this subgroup. For DSA-negative c-aABMR cases (N = 42), the level of glomerulitis tended to be associated with the different genotypes (P = 0.05), i.e. similar to that observed for the total cohort.

In addition, ptc expressed as ptc score (P = 1.00) or as extent of ptc, i.e. being focal (< 50%) versus diffuse (≥ 50%) (P = 0.96) was not associated with *FCGR3A* 158 F/V-genotypes and this was also not observed for DSA-negative cases.

Positivity of C4d within PTC is associated with complement activation, which represent another mechanism by which DSAs can inflict damage to the renal allograft^1,7^. In our study, positivity for C4d was only detected in 21% of the cases diagnosed with c-aABMR (Table 1) and although associated with presence of DSAs (P = 0.001), no association with extent of microvascular inflammation was observed (P = 0.28). Moreover, *FCGR3A* 158 F/V-genotypes were not associated to C4d-positivity (P = 0.57), which might relate to the fact that they represent different mechanisms of mediating damage to the graft.

### The *FCGR3A* 158 V/V-genotype is associated with decreased renal allograft survival

**Figure 3:**
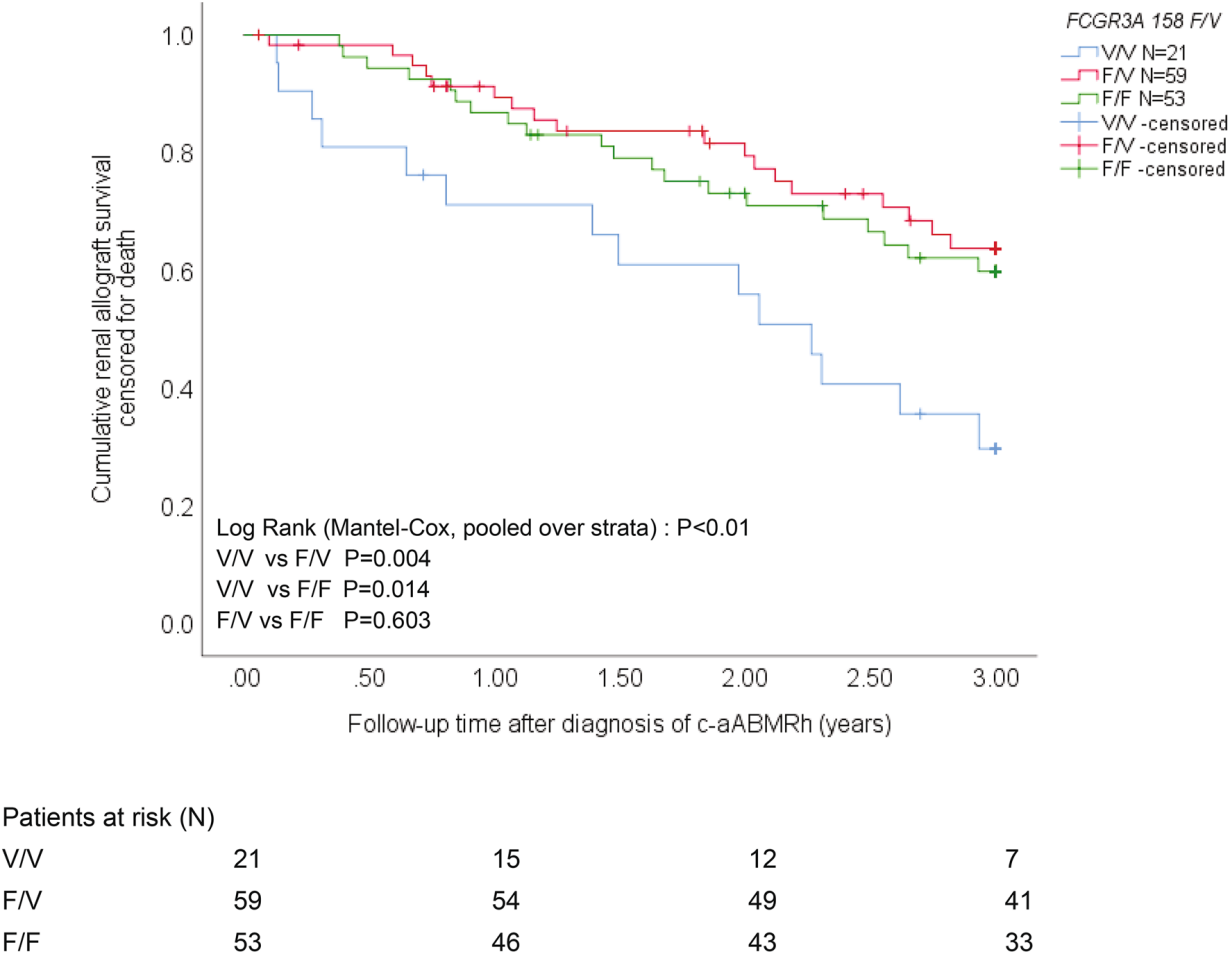
*FCGR3A* 158 F/V-genotype and renal allograft survival in c-aABMRh kidney transplant recipients. The c-aABMR-related allograft survival censored for death is depicted for the different *FCGR3A* 158 F/V-genotypes. Overall pooled over strata P value and P values for the difference between the different strata (genotypes) were calculated by Mantel–Cox log-rank statistical analysis. P values < 0.05 were considered statistically significant (blue, red and green lines represent allograft survival for V/V-, F/V- and F/F-genotype, respectively). At start and at 1, 2 and 3 years of follow-up, the number of kidney transplant recipients at risk are depicted for the different *FGCR3A* 158 F/V-genotypes.

In order to evaluate whether this single nucleotide polymorphism within *FCGR3A* (158 F/V) is associated with renal allograft survival, Kaplan–Meier curves for the different *FCGR3A* 158 F/V-genotypes were generated for the c-aABMRh cohort. We evaluated survival of renal allografts at 3 years following diagnosis of c-aABMRh. Fifty-two out of 133 (39%) c-aABMRh kidney transplant recipients lost their renal allograft due to graft failure and 6 out of 133 (5%) died with a functioning renal allograft (Table 3). The presence or absence of DSAs was not associated with graft survival (P = 0.70) as was reported before by our group^14^ and others^22–25^. Overall renal allograft survival was associated with the *FCGR3A* 158 F/V genotype (P < 0.01; Fig. 3) and similar results were obtained for DSA-negative cases (P = 0.04). The V/V-genotype showed a significant lower renal allograft survival compared to the F/V- as well as the F/F-genotype, using the pairwise between strata comparison (Mantel–Cox log rank statistical analysis) for the total cohort (P < 0.01 and P = 0.01, respectively) as well as DSA-negative cases (P = 0.03). Median (min–max) renal allograft survival times amounted to 2.1 (0.1–3), 2.7 (0.1–3) and 3.0 (0.4–3) years after diagnosis of c-aABMRh for the *FCGR3A* 158 V/V-, F/V- and F/F-genotype, respectively. Graft survival was not affected by the *FCGR3A* 158 F/V-genotype for the control cohort of kidney transplant recipients without rejection (P = 0.90; data not shown).

**Table 3:** *FCGR3A* 158 F/V genotypes of c-aABMRh kidney transplant recipients and survival.

	c-aABMRh^+^ kidney transplant recipients				
	Overall (N = 133)	V/V(N = 21)	F/V (N = 59)	F/F (N = 53)	*P* value
**Kaplan Meier survival 3 years after c-aABMRh diagnosis**					
% patient survival	95	95	91	100	0.01
% death-censored graft survival	61	33	69	62	< 0.01
% overall graft survival	56	29	61	62	< 0.01

To determine whether the V/V-genotype was an independent factor contributing to the risk of renal allograft loss, relevant clinical characteristics from Table 1, like age of the kidney transplant recipient at transplantation, gender of kidney transplant recipient, age of the kidney donor, type of kidney donor (living or deceased and ABO-compatible or ABO-incompatible), re-transplantation and eGFR at time of the for-cause biopsy were included in a multivariate Cox regression model. Out of these variables only the *FCGR3A* 158 V/V-genotype (P = 0.04) together with eGFR at time of diagnosis (HR 0.94, 95% CI 0.91–0.96, P < 0.01) were independently associated with renal allograft survival). Chronic-aABMRh kidney transplant recipients having the *FCGR3A* 158 V/V-genotype had a 1.98-fold (95% CI 1.01–3.82) increased risk (P = 0.04) for losing their allograft within 3 years.

## Discussion

In this study, we investigated the association between a functional *FCGR3A* 158 F/V single nucleotide polymorphism and diagnosis of c-aABMR and renal allograft loss thereafter. The results show that the V-allele was linked to increased expression of CD16 on NK cells and CD16-mediated NK cell cytotoxic potential. In addition, this allele was associated with an increased glomerulitis score and the V/V-genotype was associated with a decreased renal allograft survival after the diagnosis of c-aABMR. However, the *FCGR3A* 158 V/V-genotype appeared not to be a risk factor per se for the development of c-aABMR after transplantation.

This observation adds evidence for the pathogenicity of the V-allele as was shown in a previous publication that studied the impact of *FCGR3A* 158 F/V genotypes in kidney transplant recipients with c-aABMR^16^. In accordance with our study, the distribution of the F/V alleles was similar to the control population. Moreover, the V-allele was associated with a higher degree of peritubular capillarities (PTC) but did not affect the slope of eGFR loss and graft loss. As discussed by the authors, the latter finding is counterintuitive and contradictory to earlier studies, which showed a relation between the degree of MVI and graft loss^26–28^. In our cohort, we found a higher glomerulitis score to be associated with the V-allele of the *FCGR3A* 158 F/V single nucleotide polymorphism.

The V-allele was associated with increased levels of CD16 and CD16-mediated NK function as assessed by the CD16 DRI and CD107a URI. The V/V-genotype, but not the F/V-genotype was correlated with an increased risk for renal allograft loss. Unlike our study, where for-cause biopsies were taken, protocol biopsies were taken in the study by Arnold et al.^16^, allowing identification of cases with subclinical c-aABMR that may have a much better prognosis^29^. This may be an explanation for the discrepancy in clinical outcome associated with the *FCGR3A* 158 F/V genotypes between their cohort and ours. Furthermore, the fact that cases with c-aABMR having the V/V- or F/F-genotype tended to be more similar in baseline characteristics when compared to those having the F/V-genotype, could be another explanation. Nevertheless, we observed a significant increased risk for renal allograft for cases having the V/V-genotype compared to both those having the F/V- as well as F/F/-genotype.

Overall, the findings of this study indicate that the V/V variant allows for increased activation of the NK cell after FCGR3A ligation which may cause more inflammation and progressively more tissue damage to the renal allograft with c-aABMR. The possible pathogenetic role of CD16 is underlined by the finding of increased transcript levels of CD16 in biopsies diagnosed with ABMR^8–10^.

A review recently, published by Miyairi^30^, summarizes literature on the role of NK cells as critical effectors during ABMR. Detection of NK cell infiltration in biopsies is limited by the number of specific markers and staining antibodies available. CD16 was used to stain for NK cells in lung grafts^31^, CD56+ NK cells were detected in peritubular microvasculature during ongoing biopsy-indicated ABMR^8^ and others have used CD57 and NKp46 to identify NK cells in biopsies of ongoing AMR and describe their association with microvascular inflammation^32–34^. Interestingly, activation of NK cells in absence of DSAs also strongly associated with severity of glomerulitis and peritubular capillarities and less with C4d deposition^35^. Recently, infiltrates in biopsies of kidney transplant recipients diagnosed with c-aABMR were immunophenotypically characterized^36,37^. NK cells were found at relatively low frequencies but increased proportions of NK cells within the intravascular glomerular as well as peritubular compartment were observed in cases with ABMR when compared to TCMR.

Therefore, present data suggest that NK cells and in particular the FCGR3A (CD16)^+^ are most likely involved in the pathogenesis of c-aABMR.

Of note is that myeloid cells like macrophages also express CD16 and they are among the cells found in the renal microvasculature and interstitium in cases of c-aABMR^37^. Increased macrophage-associated transcripts were described for individuals with the V-allele compared to the F/F-genotype in the ABMR cases by Arnold et al.^16^. This implies that in addition to NK cells, expression of the V/V variant of *FCGR3A* by myeloid cells may also contribute to increased inflammation and damage as CD16 appears to be indispensable for ADCC by monocytes^38^.

Several other studies have investigated the *FCGR3A* polymorphism in solid organ transplantation other than kidney transplantation. In heart transplantation, Paul et al. demonstrated that this polymorphism contributed to an early (non-invasive) evaluation of risk stratification for cardiac allograft vasculopathy (CAV), an important cause of late mortality after heart transplantation^12^. These clinical data are supported by the observation that increased expression of CD16 and CD16-mediated NK function associated with the V/V-variant were observed heart transplant recipients^12^ as well as renal transplant recipients^16^. In accordance with these findings we previously showed increased CD16 expression by circulating NK cells in cases with c-aABMR^11^. This *FCGR3A* polymorphism was also found to stratify patients at risk for acute rejection in the first 3 months after transplantation, in a cohort of lung transplant recipients^39^. They also observed the V/V-genotype to behave different from F/V-genotype, i.e. to significantly reduce the acute-rejection free survival when compared to F/V- and F/F-genotype. Taken together, the current data suggest that *FCGR3A* polymorphism is associated with rejection-related complications across various types of solid organ transplantation.

One of the limitations of our retrospective study involves the lack of DSAs from the complete c-aABMR cohort. Although DSA-positive and -negative c-aABMR are similar with respect to renal allograft survival, there might be differences in terms of underlying mechanisms of damage to the renal allograft. However, in our cohort, we did observe similar findings for the DSA-negative cohort compared to the total cohort with respect to this *FCGR3A* 158 F/V SNP and level of microvascular inflammation as well as renal allograft survival. Furthermore, antibodies directed to non-HLA might also contribute to damage to the renal allograft but this information is lacking. Another limitation of the present study is the lack of a validation cohort, allowing confirmation of our findings in a similar large group of c-aABMR cases.

Concluding, the V-allele of the *FCGR3A* 158 F/V single nucleotide polymorphism leads to increased CD16 expression and upregulated cytotoxicity of NK cells. Clinically this is associated with increased microvascular inflammation in the glomerulus and significantly decreased renal allograft survival of grafts diagnosed with c-aABMR for cases having the V/V-genotype. Assessment of this *FCGR3A* 158 F/V SNP may be of importance with respect to interpretation of results from studies. Furthermore, it may add to the risk stratification for graft loss in kidney transplant recipients by identifying patients that might benefit from a more intense immunosuppressive regime.

## Material and methods

### Study population

For this study we included 141 kidney transplant recipients, diagnosed with clinical c-aABMR (within our hospital) in the period from 1998 to 2019 of which snap-frozen PBMCs prior to transplantation were stored in the kidney transplant biobank. Patients in whom c-aABMR was diagnosed following a transplantectomy, were excluded from analysis (N = 8). As a control population we selected 116 kidney transplant recipients from the biobank, transplanted within the same period, without a diagnosis of c-aABMR. All kidney transplantations were performed across a negative CDC crossmatch. Demographic and clinical parameters were collected for the c-aABMR cohort at time of transplantation and diagnostic biopsy. All renal biopsies were done on indication (eGFR loss or proteinuria) and re-evaluated by an experienced renal pathologist based on the 2015 Banff classification^40^. Donor-specific anti-HLA antibodies (DSAs) were measured using the Lifecodes Lifescreen Deluxe (LMX) kit according to the manufacturer’s manual (Immunocor Transplant Diagnostics Inc., Stamford, CT, USA). Samples positive for either HLA class I (HLA-A, -B or -C) or HLA class II (HLA-DQ or -DR), were further characterized with the Luminex Single Antigen assay, using LABscreen HLA class I and II antigen beads (One Lambda, Canoga Park, GA, USA). Before 2009, DSAs were not routinely assessed but 40% of the tested cases (N = 70) were DSA-positive. When histologic criteria were met, a diagnosis of c-aABMRh, termed suspicious for c-aABMR in the Banff 2015 criteria, was made in accordance with recent publications^13,14,40–42^.

Renal allograft function was determined by calculating the estimated glomerular filtration rate (eGFR) using the CKD-EPI formula.

Follow up of c-aABMRh kidney transplant recipients was until 1st of January 2020 and graft loss/failure was defined as the need for dialysis or a re-transplantation. The date of diagnosis of c-aABMRh and date of graft failure were used to calculate the graft survival upon diagnosis.

Kidney transplant recipients gave written informed consent and the study was approved by the Medical Ethical Committee of the Erasmus MC (MEC-2012-022 for pre-transplant material and MEC-2015-222 for the collection of data from cases of c-aABMRh and controls). The study was conducted in accordance with the Declaration of Helsinki and the Declaration of Istanbul.

### Peripheral blood mononuclear cell isolation

Prior to and at time of the for-cause biopsy, peripheral blood mononuclear cells (PBMCs) were isolated from heparinized blood samples by using Ficoll-Paque (GE HealthCare, Uppsala, Sweden). Two million PBMCs of the sample, obtained prior to transplantation, were snap-frozen in liquid nitrogen for isolation of genomic DNA (see below). The remaining PBMCs were washed, frozen at 10 × 10^6^/vial in RPMI-1640 with Glutamax (ThermoFisher Scientific, Landsmeer, The Netherlands) supplemented with 100 IU/mL penicillin/streptomycin and 10% heat-inactivated pooled serum and 10% dimethyl sulphoxide (Sigma Aldrich, Darmstadt, Germany) in liquid nitrogen until further use.

### *FCGR3A* 158 F/V (rs396991) genotyping

Genomic DNA was extracted from 2 × 10^6^ snapfrozen PBMC using the QIAamp DNA Mini isolation kit (Qiagen, Venlo, The Netherlands) according to manufacturer’s instruction. *FCGR3A* rs396991 genotype was determined by the StepOnePlus Real-Time PCR detection system (Applied Biosystems, Darmstadt, Germany) using Taqman SNP Genotyping assay (assay ID C_25815666_10; ThermoFisher Scientific Inc, Bleiswijk, The Netherlands) and Taqman Universal PCR Master Mix according to manufacturer’s instruction.

### Natural killer (NK) cell function

The CD16-mediated NK cell function was evaluated by adapting the NK-CHAT as described Legris et al.^43^. Briefly, 10^6^ allogeneic target cells (Raji, ATCC CCL-86) were carboxyfluorescein diacetate succinimidyl ester (CFSE; Molecular Probes, Bleijswijk, The Netherlands)-labeled according to manufacturer’s instruction. These target cells were co-cultured at a 1:1 ratio with PBMCs of kidney transplant recipients genotyped for *FCGR3A* 158 F/V (N = 35), allophycocyan (APC)-labelled anti-CD107a (Becton Dickinson; BD, Erembodegem, Belgium) and a cytokine secretion inhibitor (EBioscience, Bleijswijk, The Netherlands) for 3 h. The co-culture was performed with or without anti-human CD20-human-IgG1 (10 μg/mL; InvivoGen Toulouse, France) to evaluate CD16-dependent NK-cell function. As a control, PBMCs were left unstimulated to determine baseline median fluorescence intensity (MFI) of CD16 expression by circulating NK cells. Upon 3 h of stimulation, cells were harvested and stained for dead cells using Fixeable Viability Stain (FVS)-780 (BD). Following a wash, the cell surface was stained for 30 min at room temperature using the following monoclonal antibodies to identify NK cells (phycoerythrin (PE)-labeled anti-CD16/PerCPCy5.5 (peridinin-chlorophyll protein (PerCP)-Cy5.5-labeled anti-CD56, both from BD) and exclude unwanted cells like T cells (brilliant violet (BV)510-labeled anti-CD3, BD) and monocytes (APC-H7-labeled anti-CD14, BD). The following parameters were determined to evaluate CD16-mediated NK cell function: (1) CD16 downregulation index (DRI, ratio MFI CD16 in absence of anti-hCD20-hIgG1 over MFI in presence of anti-hCD20-hIgG1) and (2) CD107a upregulation index (URI, ratio % CD107a^+^ NK cells in presence of anti-hCD20-hIgG1 over %CD107a^+^ NK cells in absence of anti-hCD20-hIgG1). CD107a (lysosomal-associated membrane protein; LAMP-1) is a marker identifying degranulation of NK cells which is highly correlated to their cytotoxic potential^44^. NK cells were identified as CD3^-^CD14^-^ and CD56^+^.

Samples were measured on the FACSCanto II (BD; 3 laser, 8 color configuration 4:2:2) and analyzed using Kaluza software version 2.1 (Beckman Coulter BV., Woerden, The Netherlands). We acquired at least 10,000 NK cells.

### Statistical analyses

Normally distributed data are expressed as mean ± SD, non-normally distributed data as median and IQR. Continuous variables of c-aABMRh and control kidney transplant recipients were compared using unpaired T test or Mann–Whitney U test. Discrete data were analyzed as frequencies with Chi-square test or Fisher’s exact test. Demographic as well as patient characteristics are depicted as median and IQR, number and proportion of total, respectively. Comparisons of characteristics between the different *FCGR3A* 158 F/V-genotypes was done using Chi-square test for discrete data and Kruskal–Wallis for continuous data. Death-censored graft survival was assessed taking into account the *FCGR3A* 158 F/V-genotype for the c-aABMRh cohort by Kaplan–Meier analysis with log-rank statistics for difference between strata (pooled and pairwise). Multivariate Cox regression analysis, using the Enter method as well as the Forward and Backward stepwise Likelihood Ratio method, was used to evaluate the significance and contribution of the *FCGR3A* 158 F/V-genotype and other relevant clinical characteristics with respect to renal allograft survival. The level of glomerulitis (g) and peritubular capillaritis (ptc) as well as the combination thereof representing the total level of microvascular inflammation (MVI) were correlated to the *FCGR3A* 158 F/V-genotype. Statistical analyses were performed using GraphPad Prism 5 software (GraphPad Software La Jolla, CA,USA) and IBM SPSS statistics for Windows, version 25 (SPSS Inc. IL, USA). The significance level (P value) was two-tailed and an α of 0.05 was used for all analyses.

## Data Availability

The datasets generated during and/or analysed during the current study are available from the corresponding author on reasonable request.

## Abbreviations

ABO: Blood group ABO
ADCC: Antibody-dependent cellular cytotoxicity
APC(-H7): Allophycocyanin(-H7)
BV: Brilliant violet
c-aABMR(h): Chronic active antibody-mediated rejection (with histopathologic features only)
CAV: Cardiac allograft vasculopathy
CD: Cluster of differentiation
CFSE: Carboxyfluorescein succinimidyl ester
DRI: Downregulation index
DSAs: Donor-specific antibodies
eGFR: Estimated glomerular filtration rate
FCGR: Fc gamma receptor
FVS780: Fixeable viability stain-780
HLA: Human leukocyte antigen
IgG: Immunoglobulin G
IQR: Interquartile range
MFI: Median fluorescence intensity
NK: Natural killer
NK-CHAT: Natural killer-cellular humoral activation test
PE: Phycoerythrin
PerCPCy5.5: Peridinin-chlorophyll protein-Cy5.5
PMN: Polymorphonuclear neutrophils
PRA: Panel reactive antibodies
SNP: Single nucleotide polymorphism
URI: Upregulation index

## References

[1] Racusen LC (2003). Antibody-mediated rejection criteria—an addition to the Banff 97 classification of renal allograft rejection. Am. J. Transplant..

[2] Racusen LC (1999). The Banff 97 working classification of renal allograft pathology. Kidney Int..

[3] Michielsen LA, van Zuilen AD, Krebber MM, Verhaar MC, Otten HG (2016). Clinical value of non-HLA antibodies in kidney transplantation: Still an enigma?. Transplant. Rev. (Orlando).

[4] Loupy A, Lefaucheur C (2018). Antibody-mediated rejection of solid-organ allografts. N. Engl. J. Med..

[5] Castro-Dopico T, Clatworthy MR (2016). Fcgamma receptors in solid organ transplantation. Curr. Transplant Rep..

[6] Sis B, Halloran PF (2010). Endothelial transcripts uncover a previously unknown phenotype: C4d-negative antibody-mediated rejection. Curr. Opin. Organ. Transplant..

[7] Haas M (2014). Banff 2013 meeting report: Inclusion of c4d-negative antibody-mediated rejection and antibody-associated arterial lesions. Am. J. Transplant..

[8] Hidalgo LG (2010). NK cell transcripts and NK cells in kidney biopsies from patients with donor-specific antibodies: Evidence for NK cell involvement in antibody-mediated rejection. Am. J. Transplant..

[9] Hidalgo LG (2012). Interpreting NK cell transcripts versus T cell transcripts in renal transplant biopsies. Am. J. Transplant..

[10] Venner JM, Hidalgo LG, Famulski KS, Chang J, Halloran PF (2015). The molecular landscape of antibody-mediated kidney transplant rejection: Evidence for NK involvement through CD16a Fc receptors. Am. J. Transplant..

[11] Sablik KA, Litjens NHR, Klepper M, Betjes MGH (2019). Increased CD16 expression on NK cells is indicative of antibody-dependent cell-mediated cytotoxicity in chronic-active antibody-mediated rejection. Transpl. Immunol...

[12] Paul P (2018). Genetic and functional profiling of CD16-dependent natural killer activation identifies patients at higher risk of cardiac allograft vasculopathy. Circulation.

[13] Sablik KA, Clahsen-van Groningen MC, Damman J, Roelen DL, Betjes MGH (2019). Banff lesions and renal allograft survival in chronic-active antibody mediated rejection. Transpl. Immunol..

[14] Sablik KA (2018). Chronic-active antibody-mediated rejection with or without donor-specific antibodies has similar histomorphology and clinical outcome—a retrospective study. Transpl. Int..

[15] Sablik KA (2019). Treatment with intravenous immunoglobulins and methylprednisolone may significantly decrease loss of renal function in chronic-active antibody-mediated rejection. BMC Nephrol..

[16] Arnold ML (2018). Functional Fc gamma receptor gene polymorphisms and donor-specific antibody-triggered microcirculation inflammation. Am. J. Transplant..

[17] de Weerd AE, Betjes MGH (2018). ABO-incompatible kidney transplant outcomes: A meta-analysis. Clin. J. Am. Soc. Nephrol..

[18] Torkildsen O (2005). Ethnic variation of Fc gamma receptor polymorphism in Sami and Norwegian populations. Immunology.

[19] Lehrnbecher T (1999). Variant genotypes of the low-affinity Fcgamma receptors in two control populations and a review of low-affinity Fcgamma receptor polymorphisms in control and disease populations. Blood.

[20] Dourado MEJ, Ferreira LC, Freire-Neto FP, Jeronimo SM (2016). No association between FCGR2A and FCGR3A polymorphisms in Guillain–Barre Syndrome in a Brazilian population. J. Neuroimmunol..

[21] Loupy A (2014). Molecular microscope strategy to improve risk stratification in early antibody-mediated kidney allograft rejection. J Am Soc Nephrol.

[22] De Serres SA (2016). 2013 Banff criteria for chronic active antibody-mediated rejection: Assessment in a real-life setting. Am. J. Transplant..

[23] Halloran PF, MerinoLopez M, BarretoPereira A (2016). Identifying subphenotypes of antibody-mediated rejection in kidney transplants. Am. J. Transplant..

[24] Lesage J (2015). Donor-specific antibodies, C4d and their relationship with the prognosis of transplant glomerulopathy. Transplantation.

[25] Patri P (2016). Development and validation of a prognostic index for allograft outcome in kidney recipients with transplant glomerulopathy. Kidney Int..

[26] Kozakowski N (2015). The diffuse extent of peritubular capillaritis in renal allograft rejection is an independent risk factor for graft loss. Kidney Int..

[27] Lerut E, Naesens M, Kuypers DR, Vanrenterghem Y, Van Damme B (2007). Subclinical peritubular capillaritis at 3 months is associated with chronic rejection at 1 year. Transplantation.

[28] Loupy A (2009). Outcome of subclinical antibody-mediated rejection in kidney transplant recipients with preformed donor-specific antibodies. Am. J. Transplant..

[29] Parajuli S (2019). Subclinical antibody-mediated rejection after kidney transplantation: Treatment outcomes. Transplantation.

[30] Miyairi S, Baldwin WM, Valujskikh A, Fairchild RL (2021). Natural killer cells: Critical effectors during antibody-mediated rejection of solid organ allografts. Transplantation.

[31] Fildes JE (2008). Natural killer cells in peripheral blood and lung tissue are associated with chronic rejection after lung transplantation. J. Heart Lung Transplant..

[32] Dos Santos DC, Saraiva Camara NO, David DSR, Malheiros D (2017). Expression patterns of CD56+ and CD16+ cells in renal transplant biopsies with acute rejection: Associations with microcirculation injuries and graft survival. Nephrology (Carlton).

[33] Kildey K (2019). Specialized roles of human natural killer cell subsets in kidney transplant rejection. Front. Immunol...

[34] Shin S (2015). Interpreting CD56+ and CD163+ infiltrates in early versus late renal transplant biopsies. Am. J. Nephrol..

[35] Callemeyn J (2020). Transcriptional changes in kidney allografts with histology of antibody-mediated rejection without anti-HLA donor-specific antibodies. J. Am. Soc. Nephrol..

[36] Calvani J (2020). In situ multiplex immunofluorescence analysis of the inflammatory burden in kidney allograft rejection: A new tool to characterize the alloimmune response. Am. J. Transplant..

[37] Sablik KA, Jordanova ES, Pocorni N, Clahsen-van Groningen MC, Betjes MGH (2019). Immune cell infiltrate in chronic-active antibody-mediated rejection. Front. Immunol..

[38] Yeap WH (2016). CD16 is indispensable for antibody-dependent cellular cytotoxicity by human monocytes. Sci. Rep..

[39] Paul P (2019). FCGR3A and FCGR2A genotypes differentially impact allograft rejection and patients' survival after lung transplant. Front. Immunol..

[40] Loupy A (2017). The Banff 2015 kidney meeting report: Current challenges in rejection classification and prospects for adopting molecular pathology. Am. J. Transplant..

[41] Senev A (2019). Histological picture of antibody-mediated rejection without donor-specific anti-HLA antibodies: Clinical presentation and implications for outcome. Am. J. Transplant..

[42] Haas M (2018). The Banff 2017 Kidney Meeting Report: Revised diagnostic criteria for chronic active T cell-mediated rejection, antibody-mediated rejection, and prospects for integrative endpoints for next-generation clinical trials. Am. J. Transplant..

[43] Legris T (2016). Antibody-dependent NK cell activation is associated with late kidney allograft dysfunction and the complement-independent alloreactive potential of donor-specific antibodies. Front. Immunol..

[44] Tomescu C, Chehimi J, Maino VC, Montaner LJ (2009). Retention of viability, cytotoxicity, and response to IL-2, IL-15, or IFN-alpha by human NK cells after CD107a degranulation. J. Leukoc. Biol..

